# Nucleic Acid Sensors and Programmed Cell Death

**DOI:** 10.1016/j.jmb.2019.11.016

**Published:** 2020-01-17

**Authors:** Jonathan Maelfait, Layal Liverpool, Jan Rehwinkel

**Affiliations:** 1 VIB Center for Inflammation Research, 9052 Ghent, Belgium; 2 Department of Biomedical Molecular Biology, Ghent University, 9052 Ghent, Belgium; 3 Medical Research Council Human Immunology Unit, Medical Research Council Weatherall Institute of Molecular Medicine, Radcliffe Department of Medicine, University of Oxford, Oxford OX3 9DS, UK

**Keywords:** Apoptosis, Pyroptosis, Necroptosis, Type I interferon, Nucleic acid sensing

## Abstract

Nucleic acids derived from microorganisms are powerful triggers for innate immune responses. Proteins called RNA and DNA sensors detect foreign nucleic acids and, in mammalian cells, include RIG-I, cGAS, and AIM2. On binding to nucleic acids, these proteins initiate signaling cascades that activate host defense responses. An important aspect of this defense program is the production of cytokines such as type I interferons and IL-1β. Studies conducted over recent years have revealed that nucleic acid sensors also activate programmed cell death pathways as an innate immune response to infection. Indeed, RNA and DNA sensors induce apoptosis, pyroptosis, and necroptosis. Cell death *via* these pathways prevents replication of pathogens by eliminating the infected cell and additionally contributes to the release of cytokines and inflammatory mediators. Interestingly, recent evidence suggests that programmed cell death triggered by nucleic acid sensors plays an important role in a number of noninfectious pathologies. In addition to nonself DNA and RNA from microorganisms, nucleic acid sensors also recognize endogenous nucleic acids, for example when cells are damaged by genotoxic agents and in certain autoinflammatory diseases. This review article summarizes current knowledge on the links between nucleic acid sensing and cell death and explores important open questions for future studies in this area.

## Highlights

• Nucleic acid sensors detect foreign or unusual DNA and RNA.
• DNA/RNA sensing triggers innate immune responses, including programmed cell death.
• Nucleic acid sensors induce apoptosis, necroptosis, and pyroptosis.
• Programmed cell death eliminates infected cells and contributes to inflammation.
• These responses are highly cell type and species specific.

## Graphical abstract

**Figure:**
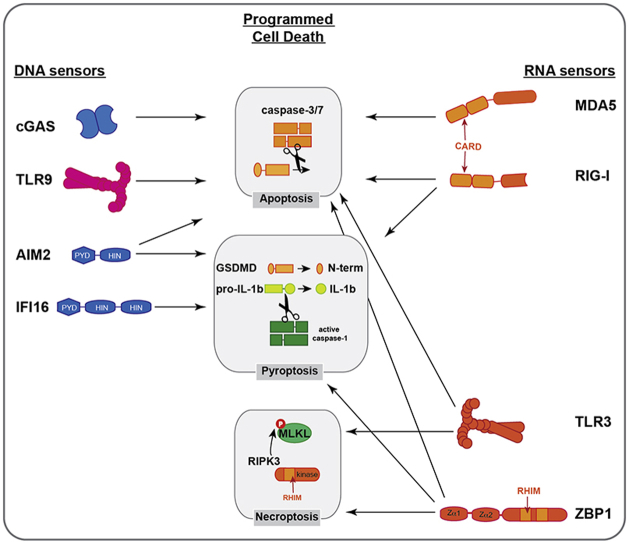

## Introduction

DNA and RNA play fundamental roles in the immune system [1]. Unusual and aberrantly localized nucleic acids are molecular signatures indicative of the presence of microorganisms or of perturbations to cellular homeostasis [2,3]. A variety of mammalian, germ-line encoded proteins, called nucleic acid sensors, bind directly to such DNA and RNA species and then signal for the induction of host defense responses. Examples are the Toll-like receptors (TLRs) 3, 7, 8, and 9, the RIG-I-like receptors (RLRs), cyclic guanosine-monophosphate adenosine-monophosphate synthase (cGAS), and absent in melanoma 2 (AIM2). These proteins have been well studied in the context of infectious insults, particularly viruses. Being obligate intracellular pathogens, viruses need to introduce and replicate their genetic material in the cells they infect. Therefore, nucleic acid sensing is a broadly effective strategy employed by host cells to detect invading viruses. However, healthy cells contain a variety of endogenous DNA and RNA species, necessitating molecular mechanisms that prevent the activation of nucleic acid sensors in the absence of infection. These include: (i) recognition of nucleic acids in subcellular compartments usually devoid of DNA or RNA; (ii) detection of either the presence or absence of chemical modifications on viral nucleic acids; (iii) sensing of unusual nucleotide sequences or secondary and tertiary structures; (iv) recognition of changes in DNA or RNA abundance; and (v) elaborate positive and negative feedback loops regulating nucleic acid sensors [1,2]. Stress and damage of cells, as well as mutations—for example, in genes encoding nucleases—sometimes result in a breakdown of these mechanisms and subsequent sensing of endogenous nucleic acids. This is relevant in the context of autoinflammatory diseases and in cancer [[3], [4], [5]].

Once activated by DNA or RNA, nucleic acid sensors induce innate immune responses, the best characterized of which is the induction of cytokines. For example, TLRs, RLRs, and cGAS all activate signaling cascades that transcriptionally induce the genes encoding type I interferons (IFNs), which are important mediators of antiviral immunity [6]. Another example is the double-stranded (ds) DNA sensor AIM2, which nucleates the formation of an inflammasome, leading to proteolytic maturation of IL-1β and other cytokines [7]. Although cytokine production plays an essential role in nucleic acid–triggered host defense, other signaling outputs of nucleic acid sensing have been described. PKR detects dsRNA and then phosphorylates and thereby inhibits eIF2α, a translation initiation factor [8]. This results in a global shutdown of protein translation in virally infected cells, preventing the production of viral proteins. Much like PKR, OAS family proteins also detect dsRNA following virus infection [9]. This results in activation of the enzymatic function of OAS proteins to produce 2′-5′ oligoadenylate, which in turn activates RNase L. This nuclease then cleaves cellular and viral RNAs and thereby prohibits replication of RNA viruses.

Another distinct consequence of engagement of nucleic acid sensors is the activation of programmed cell death. Indeed, apoptosis, pyroptosis, and necroptosis can all be initiated by DNA and RNA sensors. These pathways are summarized in Table 1. “Suicidal” cell death can be advantageous to the host in a number of ways [[10], [11], [12]]. Firstly, it prevents replication of intracellular pathogens by elimination of infected cells. Secondly, some types of programmed cell death such as pyroptosis result in the release of cytokines, particularly IL-1β. Thirdly, when cell death involves damage to the plasma membrane, as is the case during necroptosis and pyroptosis, intracellular molecules such as ATP are released into the extracellular space and serve as damage associated molecular patterns [13]. Fourthly, cell death facilitates antigen uptake by professional antigen presenting cells, thereby eliciting adaptive immune responses [14]. Finally, cell corpses can entrap and thereby disable pathogenic microorganisms [11].

**Table 1:** Overview of apoptosis, pyroptosis, and necroptosis.

	Apoptosis	Pyroptosis	Necroptosis
Cellular events	cell shrinkage DNA fragmentation apoptotic bodies	pore formation loss of plasma membrane integrity swollen cellular organelles	
Inflammation	noninflammatory	proinflammatory	
Key proteins^a^	caspase-3	ASC caspase-1 GSDMD	RIPK3 MLKL
DNA sensors^a^	cGAS-STING TLR9	AIM2 IFI16	
RNA sensors^a^	RIG-I MDA5	NLRP3 NLRP9b	TLR3 ZBP1

a Selected examples, please see text for further details.

In this article, we discuss recent examples of how nucleic acid sensors couple to programmed cell death, focusing on some of the key questions in the field: What are the mechanisms of self versus nonself discrimination by nucleic acid sensors in the context of cell death? What are the molecules driving cell death downstream of nucleic acid sensors? What is the importance of cell death instructed by DNA and RNA sensors to host defense against pathogens, in cancer and during autoinflammation? We will first explore DNA sensors and then RNA sensors in separate chapters.

## DNA Sensors

Innate immune DNA sensors are DNA-binding proteins that detect perturbations in cellular DNA homeostasis and activate intracellular innate immune signaling cascades [15]. These perturbations can be caused by infection where foreign DNA is introduced into cells, or by damage or mislocalization of self-DNA, which is usually compartmentalized in organelles. DNA sensors activate a wide range of innate immune responses. DNA sensing is particularly important in the context of viral infection where a key innate immune response is the induction of type I IFNs, which signal in an autocrine and paracrine manner to establish an anti-viral state [6]. As such, type I IFNs not only restrict viruses in the infected cell but also limit the spread of viruses to surrounding cells. In addition to type I IFN induction, several DNA sensors and proteins involved in downstream signaling activate programmed cell death as an innate immune response to infection. This section will focus on the role of DNA sensors in the activation of programmed cell death pathways.

### AIM2

The inflammasome is a large protein complex that orchestrates oligomerization and activation of caspase-1 by auto-proteolytic cleavage. Active caspase-1 in turn cleaves and thereby matures the pro-inflammatory cytokines IL-18 and IL-1β [16]. Inflammasome activation also results in the induction of pyroptosis, an inflammatory form of cell death (Table 1). Pyroptosis involves cell swelling, lysis, and ultimately the release of cytoplasmic content [17].

**Fig. 1:**
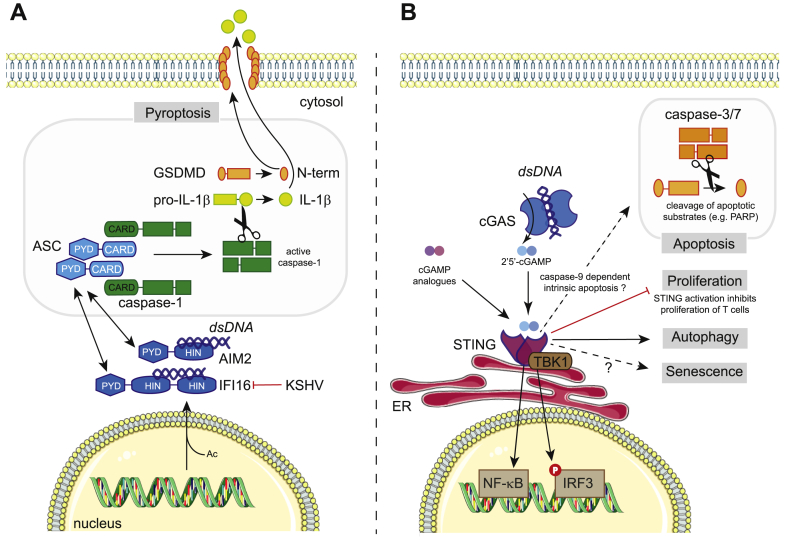
Cell death pathways triggered by DNA sensors. (A) The AIM2-like receptors AIM2 and IFI16 recognize dsDNA and induce pyroptosis. AIM2 resides in the cytosol, while IFI16 can sense viruses such as KSHV in the nucleus. Acetylation (Ac) of IFI16 promotes its nuclear export. KSHV antagonizes IFI16 by inducing its proteasomal degradation. AIM2 and IFI16 bind to dsDNA *via* their HIN200 domains (HIN) and subsequently recruit ASC *via* Pyrin domain (PYD) interactions. ASC contains a CARD domain, which interacts with the CARD of pro-caspase-1, forming a large molecular weight complex called the “ASC-speck”. Autocatalytic cleavage of procaspase-1 results in the formation of active caspase-1, which cleaves GSDMD releasing the cytotoxic N-terminal fragment (p30) that forms pores in the cell membrane. Caspase-1 also cleaves pro-IL1β, leading to the maturation into its biologically active form. (B) The cGAS/STING pathway detects the presence of dsDNA inside the cytosol and promotes antiviral immunity by inducing type I IFN production. On binding of dsDNA, cGAS synthesizes the cyclic dinucleotide cGAMP, a ligand for the ER-resident protein STING. cGAMP analogues, bacterial cyclic dinucleotides and other molecules such as DMXAA can also bind STING. Activated STING recruits the kinase TBK1, which phosphorylates the transcription factor IRF3. IRF3 then dimerizes and translocates to the nucleus where it controls type I IFN gene expression. STING also induces the activation of the pro-inflammatory transcription factor NF-κB. Engagement of cGAS/STING can also induce cell death presumably *via* the cell-intrinsic caspase-9 mediated apoptotic pathway. Active caspase-9 activates the executioner caspases 3 and 7. Other outcomes of cGAS/STING stimulation include cellular senescence, inhibition of T cell proliferation and autophagy. The signaling pathways that control these processes are poorly characterized.

AIM2 is a DNA sensor that activates the inflammasome [[18], [19], [20], [21]]. AIM2 is cytosolic and contains a pyrin domain and a HIN200 domain. HIN200 domains are positively charged and bind to dsDNA in a sequence independent manner [22]. On dsDNA binding, the pyrin domain of AIM2 interacts with the pyrin domain of the key inflammasome adaptor apoptosis-related speck-like protein (ASC). ASC then nucleates an inflammasome using its caspase activation and recruitment domain (CARD) to associate with the CARD of pro-caspase-1, triggering multimerisation and autocatalytic cleavage to release active caspase-1. This occurs in a large structure, the so-called “ASC speck” that can be readily visualized by microscopy [[23], [24], [25]]. Active caspase-1 subsequently catalyzes the processing of pro-IL-18 and pro-IL-1β into the mature cytokines (Fig. 1A). The active forms of these proinflammatory cytokines mediate downstream inflammation and immune responses once released from cells.

Pyroptosis kills cells through the action of gasdermin D (GSDMD) [26,27], a substrate cleaved by caspase-1 and other caspases. Full-length GSDMD is in an auto-inhibited, inactive conformation. Cleavage by caspase-1 leads to the release of an N-terminal GSDMD fragment (p30) that forms pores in the cell membrane to execute pyroptotic cell death [17,[26], [27], [28]]. GSDMD-mediated cytolysis is negatively regulated by the endosomal sorting complexes required for transport (ESCRT) machinery, which can initiate repair of damaged membranes [29]. GSDMD is also regulated at the transcriptional level by IRF2, which binds to a region within its gene promoter to mediate transcriptional activation [30]. IL-18 and IL-1β are not released by the canonical secretory pathway *via* the Golgi apparatus; instead, that they can exit living cells through GSDMD pores and are also released during pyroptotic cell death when the plasma membrane ruptures [[31], [32], [33], [34], [35]].

GSDMD deficiency abrogates pyroptosis in response to poly(dA:dT), a synthetic dsDNA that is sensed by AIM2 [26,27]. AIM2 has been implicated as a sensor of cytosolic DNA from invading DNA viruses and bacteria, as well as self-derived cytosolic DNA [20,36,37]. AIM2 has also been implicated in sensing of influenza A virus (IAV) infection [38]. IAV is an RNA virus. Interestingly, this study [38] demonstrates that dsDNA released into the lungs of infected mice, presumably from dying cells, is sensed by AIM2 to activate caspase-1 and IL-1β. Recognition of self-derived DNA by AIM2 is also important in the context of radiation induced DNA damage [39]. Ionizing radiation induces double-stranded breaks in DNA. AIM2 localizes to these double-stranded DNA breaks in the cell nucleus, and the resulting inflammasome activation and cell death causes tissue damage in mice [39]. Many of these studies on AIM2 employ mice or murine cells. Interestingly, in human myeloid cells, AIM2 is dispensable for DNA-mediated inflammasome activation; instead, DNA sensing *via* the cGAS-STING pathway induces NLRP3-dependent lysosomal cell death and release of IL-1β [40].

In addition to pyroptosis, AIM2 also triggers apoptosis. AIM2 is so-called because it was originally identified as a tumor suppressor, absent in melanoma [41]. Indeed, loss-of-function mutations in *AIM2* are present in more than half of the tumors from patients with bowel cancers, including colorectal cancers [42,43]. A recent study [44] demonstrated a proapoptotic and antiproliferative effect of AIM2, and further suggested that this is antagonized by the prosurvival phosphatidylinositol-3-kinase (PI3K)/protein kinase B (Akt) pathway in colorectal cancer cells. The molecular mechanisms by which AIM2 activates apoptosis in this context remain to be elucidated. AIM2 can also induce apoptosis during infection. AIM2 sensing of *Toxoplasma* infection signals the activation of a noncanonical apoptosis pathway that is GSDMD-independent and ASC- and caspase-8-dependent [45]. Future studies are needed to better understand how AIM2 drives different forms of cell death and how this is species- and cell type-dependent.

### IFI16

Much like AIM2, IFNγ-inducible protein 16 (IFI16) comprises HIN200 and pyrin domains. AIM2, IFI16 and other proteins constitute the AIM2-like receptor (ALR) family that has undergone species-specific diversification [46]. IFI16 has been suggested to function as a DNA sensor for both the induction of type I IFN [[47], [48], [49]] and the inflammasome [50]. IFI16 can be both nuclear and cytoplasmic and in its nuclear localization has been suggested to sense herpes viral genomic DNA to activate the inflammasome [[50], [51], [52]]. IFI16 becomes acetylated on recognition of viral DNA in the nucleus and is transported into the cytoplasm in complex with breast cancer 1 (BRCA1) [51]. Here, the complex activates the inflammasome by interacting with ASC to mediate caspase-1 activation and IL-1β maturation (Fig. 1A). This forms a key innate immune response to virus infection as evidenced by the fact that Kaposi's sarcoma herpes virus (KSHV) has evolved a mechanism of antagonizing this by targeting IFI16 for degradation [53].

IFI16 has further been implicated in pyroptosis of human immunodeficiency virus type-1 (HIV-1)-infected CD4 T cells [54]. Acquired immunodeficiency syndrome is caused by the progressive depletion of CD4 T cells as a consequence of HIV-1 infection. CD4 T cell depletion is associated with inflammasome formation and subsequent caspase-1 activation and pyroptotic cell death [55]. These CD4 T cells are generally quiescent by-stander cells, which primarily reside in lymphoid organs [56]. The restriction factor SAMHD1 is active in noncycling cells, including quiescent CD4 T cells, reducing their permissiveness to HIV-1 [57,58]. Nonproductive HIV-1 infection of these cells results in an accumulation of incomplete reverse transcripts in the cytosol. IFI16 was identified as a sensor of this HIV-1 DNA using an unbiased proteomic screen of tonsillar HIV-infected CD4 T cell lysates [54]. Indeed, IFI16 silencing or pharmacological inhibition of reverse transcription in resting human CD4 T cells prevents caspase-1 activation and cell death following abortive HIV-1 infection. This study further reports that IFI16 is localized in the cytosol of resting human tonsillar CD4 T cells and that IFI16 immunoprecipitates with HIV-1 Nef DNA [54].

A subsequent study [59] demonstrated that IFI16-mediated pyroptosis is dependent on the mode of viral spread. Specifically, direct cell-to-cell spread as opposed to cell-free transmission of virus is required to induce cell death of abortively infected CD4 T cells. Furthermore, neither AIM2 (see above) nor STING (see below) silencing rescues cell death in HIV-1 infected tonsillar T cells [54]. These observations suggest that cell type and differentiation state, as well as the route of virus entry, and perhaps other aspects of viral life cycles, play important roles in determining how dsDNA is sensed and the ensuing downstream consequences.

### cGAS-STING

The DNA sensor cGAS is essential for the type I IFN response to cytosolic dsDNA derived from microorganisms [15]. Active cGAS synthesizes the second messenger cyclic guanosine monophosphate-adenosine monophosphate (cGAMP) [60], hence its nomenclature, cGAMP synthase. cGAMP binds to and activates stimulator of IFN genes (STING). Active STING then engages TANK-binding kinase 1 (TBK1), which in turn phosphorylates the transcription factor IFN regulatory factor 3 (IRF3), leading to type I IFN gene transcription [15] (Fig. 1B). Interestingly, cGAS has been implicated in some autoinflammatory diseases and may therefore sense endogenous DNA in these settings [[61], [62], [63], [64], [65]]. Furthermore, the cGAS-STING pathway is known to play an important role in anticancer immunity and facilitates tumor-specific adaptive immune responses [66,67]. STING agonists have been proposed as adjuvants for vaccination and cancer therapy [68].

Several studies demonstrate that programmed cell death, particularly apoptosis, can be activated downstream of the cGAS-STING pathway. Natural and artificial STING agonists, including variants of cGAMP, induce intrinsic apoptosis in malignant B-cells [69]. This is characterized by decreased expression of the mitochondrial apoptosis inhibitor protein MCL-1 and by increased cleavage of caspases 3 and 9 as well as PARP, consistent with intrinsic apoptosis [69] (Fig. 1B). STING agonist induced apoptosis is not recapitulated by treatment with recombinant IFNβ suggesting that this is unlikely to be an indirect effect of signaling through the type I IFN receptor. In T cells, STING activation blocks proliferation and triggers cell death characterized by increased expression of proapoptotic genes [[70], [71], [72]]. Mechanistically, it has been suggested that activation of STING disrupts calcium homeostasis and activates ER stress in T cells, which on engagement of the T cell receptor undergo cell death [73]. In hepatocytes, endoplasmic reticulum stress triggers apoptosis in a STING- and IRF3-dependent manner [74,75]. It will be interesting to determine whether cGAS is required, too, or whether this represents a function of STING independent of upstream nucleic acid sensing [76]. A recent study suggested that, when mitosis is arrested, cGAS becomes activated with slow kinetics by genomic DNA, resulting in apoptosis involving a transcription-independent function of IRF3 [77]. Another piece of evidence connecting the cytosolic DNA sensing pathway to apoptosis stems from the observation that human fibroblasts undergo STING-dependent apoptosis on detection of DNA from herpes simplex virus 1 (HSV-1) and human cytomegalovirus (HCMV) [78]. Somewhat analogous to the situation in resting T cells infected with HIV, it has been suggested that reverse transcription intermediates trigger cell death in Human T Cell Leukemia Virus Type 1 (HTLV1) infected monocytes [79]. In this case, however, the type of cell death is apoptosis rather than pyroptosis and, instead of IFI16, STING is required [79].

The relationship between the cGAS-STING pathway and apoptosis is complex. It not only involves direct activation of apoptosis by cGAS and STING, but also the activation of cGAS in cells actively undergoing cell-intrinsic apoptosis. Indeed, mitochondrial DNA released into the cytosol *via* BAX and BAK-mediated permeabilization of the outer mitochondrial membrane has been shown to activate cGAS [[80], [81], [82], [83]]. However, it has been proposed that apoptotic caspases block the pathway and prevent the production of cytokines during apoptosis [80,82]. Similarly, under conditions of pyroptosis, cGAS is inactivated to prevent excessive cytokine production, which may be due to multiple mechanisms [84,85].

The cGAS-STING pathway can additionally induce necroptosis [86]. Indeed, intracellular DNA sensing by cGAS in bone marrow–derived macrophages and pharmacological STING activation *in vivo* activate type I IFN and TNF signaling pathways, which synergize to trigger RIPK3- and MLKL-driven necroptosis [86]. Moreover, cGAS-STING activation by mitochondrial DNA released into the cytosol has been suggested to amplify necroptosis *via* a TNF-dependent mechanism [87]. In addition to programmed cell death induction, the cGAS-STING pathway has also been implicated in the induction of autophagy [[88], [89], [90], [91], [92]] and in the regulation of cellular senescence [[93], [94], [95]] (Fig. 1B). It will be interesting for future studies to determine how STING signaling can toggle between transcriptional responses, different forms of cell death and autophagy.

### TLR9

Toll-like receptor 9 (TLR9) is an endosomal DNA sensor that induces MyD88-dependent transcriptional responses, mediated by NF-κB and IRF7 activation [96]. TLR9 has also been linked to cell death. TLR9 is strongly activated by DNA molecules containing unmethylated CpG dinucleotides, which are commonly found in bacteria but are usually methylated in vertebrates [97]. Stimulation of neuroblastoma cells with TLR9 ligands was found to induce apoptosis [98]. Indeed, treatment with CpG induces cell death that is characterized by cleavage of caspase-3 and -7 and, which is rescued by the pan-caspase inhibitor zVAD-FMK [98]. The authors additionally observed depolarization of the mitochondrial membrane potential consistent with intrinsic apoptosis. In nude mice with neuroblastoma, treatment with CpG prolongs long-term survival suggesting an important potential application for CpG oligonucleotides in cancer therapy [98]. The use of nude mice, which lack a fully functioning adaptive immune system, is consistent with T cell-independent cytolysis of the cancer cells. A number of other studies have linked TLR9 with apoptosis induction and revealed that TLR9 is not only proapoptotic, but also blocks apoptosis depending on the cellular context [[99], [100], [101], [102], [103]]. For instance, murine macrophages exposed to environmental stress undergo increased apoptosis and deficiency of TLR9 attenuates macrophage apoptosis and is associated with down-regulation of caspase-3 and PARP cleavage [102]. TLR9 activation has also been implicated in immunopathogenic necrosis during ischemic acute kidney injury, and in nonapoptotic hepatocyte death mediated by RIPK1 [104,105]. Altogether, these results reinforce the notion that the links between nucleic acid sensors and cell death are highly cell-type specific.

## RNA Sensors

Much like the DNA sensors discussed in the previous chapter, RNA sensors respond to foreign nucleic acids derived from invading microorganisms or to unusual endogenous RNA molecules [2]. Given the presence of normal cellular RNA in many different subcellular compartments, mechanisms of self versus nonself discrimination are particularly important for RNA sensors to avoid spontaneous responses. Here, we discuss programmed cell death as a consequence of the engagement of RNA sensors.

### RLRs

**Fig. 2:**
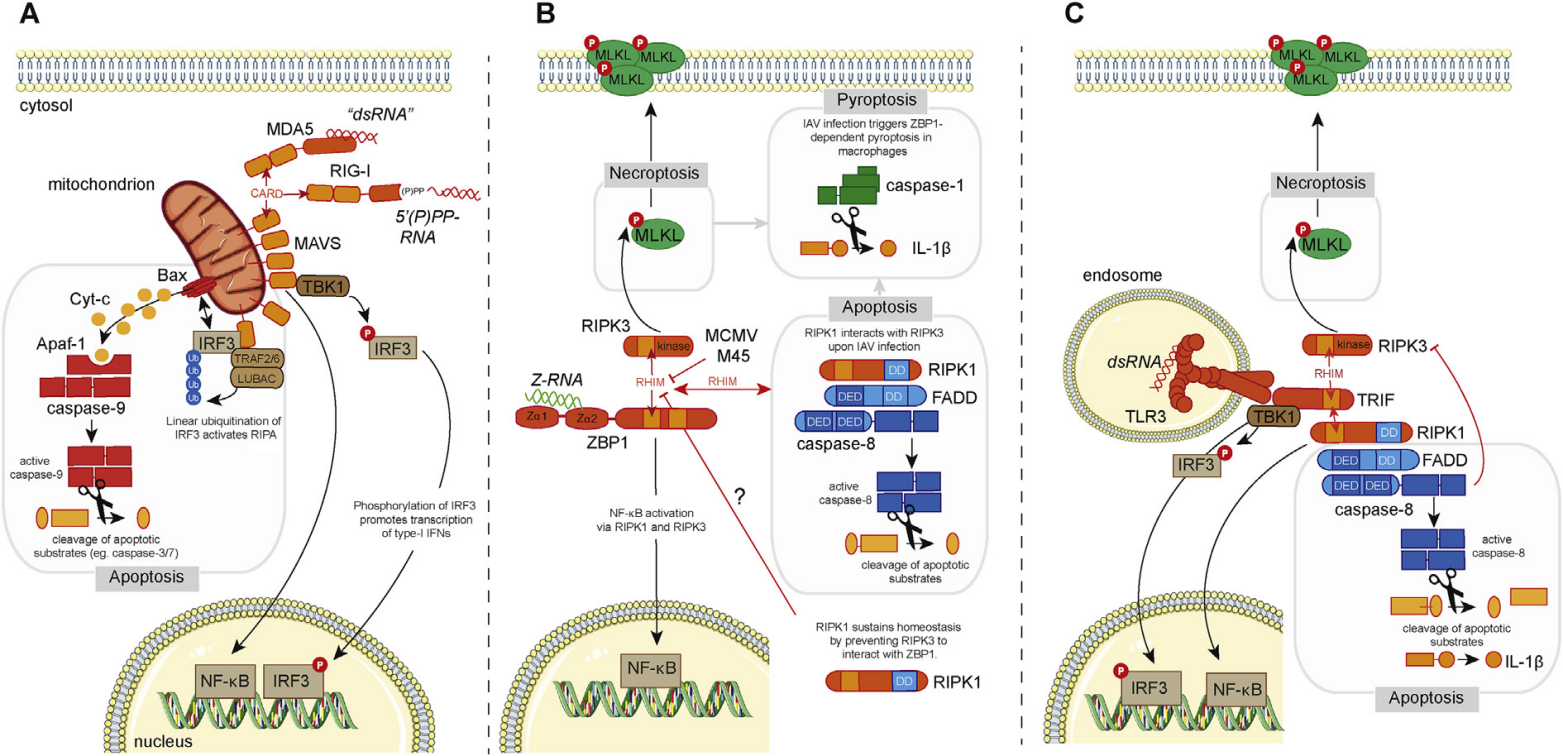
Cell death pathways triggered by RNA sensors. (A) The RLRs RIG-I and MDA5 recognize 5′ di- or triphosphorylated short stretches of base-paired RNA and long complex dsRNA, respectively. Stimulation of RIG-I and MDA5 exposes their CARDs, which interact with the CARD of MAVS. MAVS forms a multimeric complex localized at the mitochondria, from where two distinct signaling pathways diverge. One complex contains the kinase TBK1, which phosphorylates and activates the IRF3 transcription factor, which in turn controls expression type I IFN. MAVS also stimulates the activation of the proinflammatory transcription factor NF-κB. A second complex forms with TRAF2, TRAF6, and LUBAC and activates the RLR-induced IRF3 mediated pathway of apoptosis (RIPA). LUBAC adds linear ubiquitin chains onto IRF3, which activates the proapoptotic protein Bax. This leads to leakage of cytochrome c out of the mitochondria. Cytochrome c binds to and activates the proapoptotic protein Apaf-1, which induces the cleavage of the proenzyme of caspase-9 into the active form. (B) ZBP1 contains two N-terminal Z-nucleic acid binding domains (Zα1 and Zα2) through which it binds to Z-form dsRNA (Z-RNA). On activation, ZBP1 interacts with RIPK3 *via* homotypic RHIM interactions. ZBP1 contains two tandem RHIMs (RHIM-A and RHIM-B), of which only RHIM-A is required for this interaction. RIPK3 then phosphorylates MLKL, which on oligomerization triggers necroptosis by inducing permeabilization of the cell membrane. In cells infected with IAV, RIPK3 also associates with RIPK1. RIPK1 contains a death domain (DD) through which it recruits FADD. FADD associates with caspase-8 through death-effector domain (DED) interactions leading to caspase-8 activation and apoptosis. In macrophages, ZBP1-mediated necroptosis or apoptosis triggers the formation of a caspase-1 inflammasome leading to maturation of IL-1β. ZBP1 can also induce NF-κB activation and this requires RIPK1 or RIPK3. The RHIM of RIPK1 prevents ZBP1-mediated necroptosis during development and in keratinocytes. The mechanism through which RIPK1 prevents such “spontaneous” ZBP1 activation is unknown. (C) TLR3 is expressed on the cell surface (not shown) and in endosomes. The ligand-binding domain of TLR3 faces the endosomal lumen and binds to dsRNA. TLR3 signals *via* TRIF that contains a RHIM, allowing recruitment of RIPK1 and RIPK3. Interactions with RIPK1 stimulate NF-κB activation and proinflammatory gene expression. TRIF also signals *via* TBK1 and IRF3 promoting type I IFN gene expression. RIPK1 can also associate with FADD and caspase-8 to induce apoptosis. Active caspase-8 can directly cleave pro-IL1β into its biologically active form in TLR3-stimulated macrophages. When caspase-8 activation is compromised, RIPK3 is recruited to TRIF and induces necroptosis by activating MLKL, independently of RIPK1.

RIG-I-like receptors (RLRs) are a family of cytosolic RNA sensors [2]. All three members of this protein family (RIG-I, MDA5 and LGP2) have a helicase domain and a so-called C-terminal domain, both of which bind to RNA. Additionally, RIG-I and MDA5 have two N-terminal CARD domains. Binding of immunostimulatory RNAs to the CTD and helicase domains of RIG-I and MDA5 results in conformational changes that expose their CARD domains, which then interact with the CARD domain found in MAVS. This mitochondrial protein is an essential adaptor for RLRs and mediates downstream signaling, for example the induction of type I IFNs [2] (Fig. 2A). The third RLR, LGP2, lacks CARD domains and regulates the function of RIG-I and MDA5 [2].

The properties of RNA molecules that are recognized by RLRs and activation mechanisms have been studied in detail, in particular for RIG-I [106]. Using its CTD and helicase domains, RIG-I surveys the 5′-ends of RNA molecules. A number of biochemical features are detected, including (i) the presence of an uncapped triphosphate (PPP) or diphosphate (PP) group at the 5′-end of the RNA [[107], [108], [109]]; (ii) base-pairing of the 5′-(P)PP containing RNA molecule to a complementary strand of RNA [110,111]; and (iii) the absence of 2′-O methylation on the 5′-terminal nucleotide [112]. These molecular features of RIG-I-stimulatory RNAs are typically absent from the vast majority of cellular RNAs in the cytosol but are found in several viral RNA molecules, explaining selective activation in virus infected cells. In addition, some cellular noncoding RNAs normally found in the cell nucleus have the properties of RIG-I-stimulatory RNAs, and activate RIG-I when aberrantly localized in the cytoplasm of an infected cell [113,114]. The features of RNAs recognized by MDA5 are less well understood and have been reviewed recently [115].

In addition to their essential role in type I IFN induction during virus infection, RLRs have also been described to induce apoptosis. RLR activation can be triggered experimentally by cytosolic delivery of synthetic RNA agonists such as poly(I:C), which engages MDA5 and RIG-I, or 5′-triphosphate containing, base-paired RNAs that activate RIG-I [2]. Transfection of such RNAs into the cytosol of cells not only results in type I IFN production but also in the induction of apoptosis [[116], [117], [118]]. If activated naturally by virus infection, RLR-triggered apoptosis can be beneficial to the host by eliminating infected cells [119]. Interesting translational approaches have been suggested to harness RLR-mediated apoptosis to eliminate cells persistently infected with viruses and to kill cancer cells [[120], [121], [122], [123]]. Stimulation of RIG-I in breast cancer cells activates pyroptosis, in addition to extrinsic apoptosis, thereby promoting antitumor immune responses [123]. Moreover, RIG-I signaling has been implicated in the activation of RIPK3-mediated necroptosis driven by cooperative type I IFN and TNF signaling [86].

Mechanistically, apoptosis triggered by RLRs requires MAVS and IRF3, much like IFN induction [118,124]. However, MAVS has been suggested to form two functionally distinct complexes with IRF3. One mediates IRF3 phosphorylation by TBK1 and subsequent transcriptional induction of type I IFNs. The other complex additionally contains TRAF2, TRAF6, and the linear ubiquitin chain assembly complex (LUBAC) and modifies IRF3 by addition of linear ubiquitin chains [124,125]. IRF3 then translocates to mitochondria and together with the proapoptotic protein Bax triggers the cell intrinsic apoptosis pathway and cytochrome C release from mitochondria [124,125]. This pathway has been designated as “RIG-I-like receptor-induced IRF3 mediated pathway of apoptosis (RIPA)” [126] (Fig. 2A). It will be interesting to determine whether this pathway of IRF3-mediated apoptosis is specific to the RLR pathway or is also activated downstream of other nucleic acid sensors that signal to IFN induction *via* IRF3; for example, apoptosis initiated by cGAS and STING may involve a similar IRF3-dependent mechanism [77,79]. Intriguingly, MDA5 may not only be involved in triggering apoptosis but also in the execution of apoptosis, this has been suggested to involve cleavage of MDA5 [127].

### ZBP1

Z-DNA binding protein 1 (ZBP1, also known as DAI and DLM-1) is encoded by an interferon-stimulated gene and was described as an antiviral cytosolic DNA sensor inducing type I IFNs, hence its widely used name DNA-dependent activator of IFN-regulatory factors (DAI) [128]. Studies in *Zbp1*^−/−^ mice, however, did not support these findings [129]; ZBP1-deficient primary mouse embryonic fibroblasts and bone marrow-derived dendritic cells respond normally to dsDNA transfection into the cytosol and ZBP1 knockout mice show no defects in T cell-mediated and humoral immune responses when immunized with DNA vaccines, which depend on TBK1 and type I IFN signaling [129]. Moreover, overexpression of ZBP1 in HEK293T cells only modestly stimulates reporter constructs under control of promoter elements from type I IFN genes [[130], [131], [132]]. Importantly, seminal studies by the Chen lab have identified cGAS as a major cytosolic DNA sensor inducing type I IFNs and antiviral immunity [15].

These observations pointed to a different antiviral mechanism of action. Mocarski and colleagues were the first to show that ZBP1 induces necroptosis when cells are infected with the β-herpesvirus murine cytomegalovirus (MCMV) [133]. ZBP1 contains two tandem RIP homotypic interaction motifs (RHIMs) [130,131]. ZBP1 associates with and activates the kinase RIPK3 *via* RHIM-RHIM interactions, leading to phosphorylation of the pseudokinase MLKL, which on oligomerization forms pores in the plasma membrane causing necroptosis of the infected cell [134] (Fig. 2B). Interestingly, ZBP1-mediated cell death occurs independently of RIPK1 [135] setting it apart from TNF-induced necroptosis, which requires the kinase activity of RIPK1 [136]. MCMV encodes viral inhibitor of RIP kinase activation (vIRA, also known as M45), which contains its own RHIM and suppresses the execution of necroptosis by preventing ZBP1-RIPK3 interactions [133,135,137,138]. Detection by ZBP1 is important *in vivo* as replication of M45 RHIM-mutant MCMV is severely attenuated in wild-type mice, while it efficiently infects mice deficient for RIPK3 or ZBP1 [133,135].

ZBP1 also acts an innate sensor inducing cell death following Influenza A (IAV) and B virus infection [139,140]. During influenza virus infection, ZBP1 signaling is positively regulated by the transcription factor IRF1 [141]. In primary mouse embryonic fibroblasts and bone marrow–derived macrophages ZBP1 nucleates a RIPK3-containing complex that contains MLKL, triggering necroptosis, and FADD, which induces caspase-8 mediated apoptosis [139,140,142]. In LPS-primed macrophages, ZBP1 drives NLRP3 inflammasome activation, pyroptosis and IL-1β secretion [140,143] (Fig. 2B). It is not clear how both apoptosis and necroptosis coexist in cell cultures infected with IAV as these events are generally considered hierarchical and mutually exclusive [144]. Also, whether this phenomenon is regulated by viral factors that antagonize programmed cell death as is the case for MCMV remains to be determined. Two groups have independently shown that IAV replication is enhanced in ZBP1-deficient mice suggesting that ZBP1-mediated cell death is an effective antiviral strategy *in vivo* [139,140]. These studies, however, report differently on the survival outcome after challenge with a lethal dose of IAV. This discrepancy may be due to the fact that different contributions of immunopathology, host tolerance, and viral resistance are a common source of variation in IAV survival studies [145,146].

Most studies on ZBP1 so far have been conducted in the mouse system. ZBP1/RIPK3-dependent antiviral cell death in human cells is less well characterized. In an unbiased approach to determine the antiviral effects of more than 350 interferon-stimulated genes, human ZBP1 was shown to prevent replication of multiple viruses, including coxsackie B virus, sindbis virus, hepatitis C virus, and vaccinia virus [147,148]. The role of ZBP1 in vaccinia virus infection was confirmed more recently [149]. Human ZBP1 has additionally been shown to be involved in the detection of herpes viruses [[150], [151], [152], [153]] and IAV [154].

ZBP1 contains two N-terminal Zα domains (Zα1 and Zα2). Zα domains are found in a few different proteins and specifically bind to double stranded RNA or DNA in the “zig-zag” or Z-conformation [155]. IAV RNA genomes immunoprecipitate with ZBP1, and ZBP1 has been shown to colocalize with IAV viral nucleoprotein complexes [139,143]. We and others recently showed that on MCMV infection IE3-mediated transcription—but not translation—of viral genes contributes to activation of ZBP1, and that newly synthesized RNA cross-links to ZBP1 [156,157]. These observations suggest that ZBP1 is an RNA sensor, at least in the context of viral infection. Activation of ZBP1 is dependent on its Zα-domains, which specifically bind to Z-form dsRNA (Z-RNA) and not to A-form dsRNA [139,140,153,156,157]. Importantly, Zα-domains are essential *in vivo* as knock-in mice, which express ZBP1 carrying point mutations in both Zα-domains that abrogate binding to Z-nucleic acids, do not restrict M45-mutant MCMV, similar to complete ZBP1 knockout animals [157]. Under physiological conditions Z-RNA is unstable [158] and the transition of A-form dsRNA into Z-RNA in living cells requires stabilization, for example through protein-RNA interactions. This may be the case in IAV viral ribonucleoprotein complexes, where the winding of IAV genomes around viral nucleoproteins may induce the Z-conformation [159]. Alternatively, certain dsRNA helices are more prone to form Z-RNA due to their sequence content [160]. Indeed, alternating CG-, rather than AT-sequences more readily transition into Z-form nucleic acids [161]. MCMV and HCMV genomes are rich in CG-repeats and contain overlapping transcription units on both DNA strands, which give rise to viral dsRNA that may adopt the Z conformation.

In addition to its role during virus infection, ZBP1 has been shown to activate RIPK3/MLKL-mediated necroptosis in cells lacking RIPK1 [162,163]. RIPK1-deficient mice die shortly after birth as a consequence of unchecked caspase-8-dependent apoptosis and RIPK3/MLKL-mediated necroptosis [164]. The RHIM-domain of RIPK1 selectively suppresses necroptosis as embryos in which the RHIM of RIPK1 is mutated develop RIPK3/MLKL-dependent dermal inflammation, but do not exhibit typical abnormalities associated with aberrant caspase-8 activation [162,163]. Crossing these mice to ZBP1 knockout animals rescues their perinatal lethality. ZBP1-induced necroptosis also drives skin inflammation in mice in which RIPK1 expression was specifically abrogated in keratinocytes [163]. In the absence of a functional RIPK1 RHIM, ZBP1 was shown to interact with RIPK3 and induce its auto-phosphorylation; however, these studies did not detect ZBP1-RIPK1 interactions [162,163] and the precise mechanism of action of how RIPK1 prevents ZBP1 activation remains unknown. Interestingly, interferon-induced expression of ZBP1 suffices to trigger necroptosis of RIPK1 deficient cells in the absence of a viral insult [[165], [166], [167]] and blockade of caspase activity on ectopic expression of ZBP1 induces cell death [157]. These findings suggest that sensing of endogenous Z-nucleic acid species by ZBP1 may contribute to the inflammatory disease seen in RIPK1 or caspase-8 deficient mice [164].

Collectively, these observations suggest that sensing of Z-nucleic acids, including Z-RNA, by ZBP1 contributes to viral clearance *via* the induction cell death, and may also underlie the development of inflammatory pathologies.

### TLR3

Toll-like receptor 3 (TLR3) is expressed on the cell surface and in the endosomal compartment and recognizes dsRNA and the dsRNA mimic poly(I:C) [168,169]. Physiological dsRNA ligands that stimulate TLR3 are generated during virus infection and may originate from genomes of dsRNA viruses, dsRNA replication intermediates or overlapping transcripts of large DNA viruses [169,170]. TLR3 signals *via* TIR domain-containing adapter inducing IFNβ (TRIF) and, through NF-κB and IRF3, stimulates production of proinflammatory cytokines and type I IFNs [[171], [172], [173], [174]] (Fig. 2C). TRIF contains a C-terminal RHIM and interacts with RIPK1 to activate NF-κB [175]. RIPK1 can also form a complex with FADD and caspase-8 to induce apoptosis [[176], [177], [178], [179]]. In macrophages, simultaneous TLR3 stimulation and inhibition of translation results in cell death accompanied by caspase-8-mediated cleavage and release of IL-1β, independently of caspase-1 [180]. When caspase-8 activity is compromised, TLR3 signals *via* RIPK3 and MLKL, independently of RIPK1, to induce necroptosis [181,182] (Fig. 2C).

TLR3 is highly expressed in a subset of dendritic cells specialized in cross-presentation of extracellular antigens [183]. In these cells, TLR3 induces important costimulatory signals for the induction of cytotoxic T cell responses against virus-infected cells [184]. Mutations in *TLR3* or *TRIF* are associated with encephalitis after infection with HSV-1 [185,186] or IAV [187]. Furthermore, in IAV infected mice, TLR3 contributes to immunopathology [188] and triggers cell death in the lungs [189]. Cells from patients with homozygous loss-of-function mutations in RIPK1 exhibit impaired proinflammatory signaling *via* TLR3 and the TNF receptor, resulting in dysregulated cytokine secretion and a predisposition to necroptosis [190].

TLR3 has been shown to induce cell death of epithelial cells in the small intestine in response to poly(I:C) administration [191] or after release of RNA from cells damaged by ionizing radiation [192]. Endogenous dsRNA released from dying cells can act as a danger signal [193] driving both beneficial and detrimental inflammatory responses that occur during tissue damage. For example, stimulation of TLR3 by noncoding RNAs released from dying keratinocytes aids tissue repair after exposure of the skin to ultraviolet light [194] or wounding [195,196]. In contrast, TLR3 signaling contributes to tissue damage in mouse models of acute respiratory distress syndrome [197], myocardial infarction [198], and sepsis [199]. An important question for future work will be whether TLR3-mediated production of proinflammatory cytokines or TLR3-induced cell death, or both, causes these phenotypes.

In some cases, TLR3-induced cell death aggravates disease by contributing to a self-propagating cycle of inflammation. Mice deficient for the LUBAC subunit SHARPIN develop chronic proliferative dermatitis [200]. These animals develop severe skin inflammation [201] due to early TNF-induced cell death of keratinocytes in the absence of an intact LUBAC [202,203]. The release of endogenous dsRNA from dying cells subsequently triggers TLR3-mediated cell death driving skin inflammation with increasing severity. Accordingly, crossing SHARPIN-deficient mice to TLR3 knockout mice prevented cell death in the skin and ameliorated disease [189]. Interestingly, TRIF signaling was reported to contribute to skin inflammation in epidermis specific RIPK1 knockout animals, suggesting a role for TLR3 (or TLR4, another TLR that signals *via* TRIF) in autoinflammatory skin disease [204], although it appears that the relative contribution of ZBP1 in this setting is greater than that of TRIF [163,204].

Age-related macular degeneration (AMD) is a complex human disease leading to progressive retinal deterioration ultimately causing blindness [205]. Choroidal neovascularization followed by vascular leakage damages the retinal pigment epithelium (RPE). Surprisingly, small interfering RNAs (siRNAs) targeting vascular endothelial growth factor-A (VEGFA), which are clinically tested to prevent angiogenesis, act through a TLR3-mediated cell death pathway and inhibit neovascularization in a sequence independent manner [206]. Indeed, siRNAs can serve as agonists of TLR3 expressed on the cell surface of both blood vessels and lymphatic endothelial cells and induce apoptosis in these cells [207]. It should be noted that the application of siRNA-TLR3 induced cytotoxicity—at least in AMD—should be approached with caution as this process also induced the degeneration of the RPE itself [208].

Taken together, programmed cell death induced by TLR3 stimulation occurs in the forms of apoptosis and necroptosis. Sensing of viral or endogenous dsRNA by TLR3 contributes to beneficial host responses such as tissue repair and cytotoxic T cell activation, but it also triggers inflammatory disease. Finally, ligands that induce TLR3-mediated cell death can be harnessed for their therapeutic potential for example in AMD and possibly also for treatment of cancers [209,210].

## Outlook

The examples discussed in this review illustrate intimate connections between innate immune nucleic acid sensors and programmed cell death pathways. Recent work has demonstrated that cell death often rivals more classical responses such as cytokine production in terms of importance to host defense against infectious or inflammatory insults. At the same time, it has also become clear that these connections are highly complex and variable depending for example on disease context and cell type. Our understanding of how divergent signaling outcomes such as transcription of cytokine genes and cell death are coordinated is at its infancy. Posttranslational modifications such as linear ubiquitin appear to play an important role in this decision [125,189].

In addition to the three types of programmed cell death discussed here (Table 1), other cell death pathways such as NETosis, paraptosis, and oxeiptosis play important roles in host-pathogen interactions [11,211,212]. It will be interesting to study the role of nucleic acids sensors as putative upstream activators of these pathways. Moreover, the list of nucleic acid sensors coupling to cell death presented in this article is not exhaustive; for example, several NOD-like receptors have been reported to sense nucleic acids, sometimes working together with helicase proteins. This includes NLRP9b, which senses RNA in conjunction with DHX9, as well as NLRP3 that responds to IAV RNA [213,214]. It seems clear that nucleic acid sensing and cell death will remain a fertile area for future research for many years to come.
